# Assessment of Cr(VI)-Induced Cytotoxicity and Genotoxicity Using High Content Analysis

**DOI:** 10.1371/journal.pone.0042720

**Published:** 2012-08-08

**Authors:** Chad M. Thompson, Yuriy Fedorov, Daniel D. Brown, Mina Suh, Deborah M. Proctor, Liz Kuriakose, Laurie C. Haws, Mark A. Harris

**Affiliations:** 1 ToxStrategies, Katy, Texas, United States of America; 2 Thermo Fisher Scientific, Pittsburgh, Pennsylvania, United States of America; 3 ToxStrategies, Austin, Texas, United States of America; 4 ToxStrategies, Rancho Santa Margarita, California, United States of America; East Carolina University, United States of America

## Abstract

Oral exposure to high concentrations of hexavalent chromium [Cr(VI)] induces intestinal redox changes, villus cytotoxicity, crypt hyperplasia, and intestinal tumors in mice. To assess the effects of Cr(VI) in a cell model relevant to the intestine, undifferentiated (proliferating) and differentiated (confluent) Caco-2 cells were treated with Cr(VI), hydrogen peroxide or rotenone for 2–24 hours. DNA damage was then assessed by nuclear staining intensity of 8-hydroxydeoxyguanosine (8-OHdG) and phosphorylated histone variant H2AX (γ-H2AX) measured by high content analysis methods. In undifferentiated Caco-2, all three chemicals increased 8-OHdG and γ-H2AX staining at cytotoxic concentrations, whereas only 8-OHdG was elevated at non-cytotoxic concentrations at 24 hr. Differentiated Caco-2 were more resistant to cytotoxicity and DNA damage than undifferentiated cells, and there were no changes in apoptotic markers p53 or annexin-V. However, Cr(VI) induced a dose-dependent translocation of the unfolded protein response transcription factor ATF6 into the nucleus. Micronucleus (MN) formation was assessed in CHO-K1 and A549 cell lines. Cr(VI) increased MN frequency in CHO-K1 only at highly cytotoxic concentrations. Relative to the positive control Mitomycin-C, Cr(VI) only slightly increased MN frequency in A549 at mildly cytotoxic concentrations. The results demonstrate that Cr(VI) genotoxicity correlates with cytotoxic concentrations, and that H2AX phosphorylation occurs at higher concentrations than oxidative DNA damage in proliferating Caco-2 cells. The findings suggest that *in vitro* genotoxicity of Cr(VI) is primarily oxidative in nature at low concentrations. Implications for *in vivo* intestinal toxicity of Cr(VI) will be discussed.

## Introduction

Hexavalent chromium [Cr(VI)] inhalation exposure is a well-accepted risk factor for human lung cancer [1]. Oral exposure to very high concentrations of Cr(VI) in drinking water was recently shown to induce intestinal tumors in mice [2], [3]. Upon ingestion, Cr(VI) is reduced to the more inert trivalent form, Cr(III), by gastric fluids due to the low pH and presence of biomolecules and foodstuffs [4], [5]. Unreduced Cr(VI) is absorbed from the intestinal lumen via anion transporters and reduced intracellularly by low molecular weight thiols (e.g. GSH), antioxidants (e.g. ascorbate), and other molecules [6], [7]. Cr(VI) is generally unreactive toward DNA, whereas Cr(III) either itself or as binary ligands (e.g. Cr-GSH) can react with DNA. Cr(VI) reduction to intermediate forms such as Cr(V) and Cr(IV) can elicit changes in cellular redox status either through depletion of thiols and antioxidants or generation of reactive oxygen species (ROS). Thus, under various *in vitro* exposure scenarios Cr(VI) has been shown to induce a wide spectrum of genotoxic lesions [8], [9], [10], [11], [12]. In addition, recent studies indicate that continuous passage of certain cells in low concentrations of Cr(VI) *in vitro* can result in transformation to malignant cells [13], [14], [15]. It is thus important to understand the risk that Cr(VI) ingestion in drinking water may have on intestinal carcinogenesis at typical environmental exposure levels.

Despite evidence for potential genotoxic effects of Cr(VI) *in vitro*, *in vivo* evidence for genotoxicity following oral exposure is equivocal [16]. The National Toxicology Program (NTP) conducted four *in vivo* micronucleus (MN) tests in three strains of mice that were exposed to Cr(VI) in drinking water for three months and reported positive MN formation only in one of the four studies, *viz*. in transgenic strain *am3*-C57BL/6 [17]. This mouse strain contains a transgene for detecting forward and reverse mutations; however, mutation analysis was not performed [2], [17]. Similar negative MN findings were observed in other studies [18], [19]. Mice chronically exposed to very high concentrations of Cr(VI) in drinking water developed small intestinal tumors (mostly adenomas) that were detected, in all but one instance, only at study termination [2]. Histopathological analyses indicated that Cr(VI) induced intestinal damage and regenerative cell proliferation [2], [3], and such effects can be seen at carcinogenic concentrations after only 7 days of exposure [20]. In contrast, rats exposed to the same Cr(VI) drinking water concentrations did not develop intestinal damage, cell proliferation or intestinal tumors [2], [3], [17]. Together, these observations suggest that Cr(VI) was not very efficient at causing DNA mutation, malignancy or death, and that intestinal damage and hyperplasia was a major factor in the tumorigenesis in mice.

It is well accepted that cytotoxicity and regenerative hyperplasia are major contributors to carcinogenesis [21], [22], [23]. We have previously hypothesized that the mode of action (MOA) for Cr(VI)-induced carcinogenicity in the mouse small intestine is the result of cytotoxicity and hyperplasia [16] as opposed to mutagenic mechanisms proposed by others [12], [24]. Evidence for this includes redox changes and villus cytotoxicity at lower Cr(VI) concentrations than those that increase crypt hyperplasia [20], induction of Nrf2 signaling at low Cr(VI) concentrations [25], lack of MN formation in duodenal crypts after 7 or 90 days of exposure to Cr(VI) [26], as well as lack of *in vivo K-ras* codon 12 GAT mutations in the mouse duodenum after 90 days of exposure [27]. Given the preponderance of data indicating that Cr(VI) is genotoxic *in vitro*, we attempted to recapitulate the *in vivo* intestinal mucosa with an *in vitro* cell model in order to a) explore whether there are differences in response to Cr(VI) in proliferating and differentiated intestinal cells, and b) examine whether oxidative DNA damage and H2AX phosphorylation were present at non-cytotoxic concentrations.

The mucosa of the small intestine is comprised of mature differentiated villus enterocytes that are directly exposed to the intestinal lumen, and poorly differentiated proliferative enterocytes that reside in glands of Leiberkühn (i.e. crypts) below the luminal surface [28], [29]. To create an *in vitro* model of these two cell populations, the human colorectal adenocarcinoma Caco-2 cell line was grown for either 1 or 21 days, and then exposed to Cr(VI) for up to 24 hours. In short-term culture, Caco-2 cells are undifferentiated and proliferating, and thus closely resemble intestinal crypt epithelial cells. Although Caco-2 cells originate from the colon, when grown to post-confluency (∼21 days) they spontaneously differentiate and develop morphological characteristics of the small intestine including polarity, intercellular junctions, microvilli, and express markers for mature enterocytes such as brush border hydrolases; as such, Caco-2 are a well-accepted model for studying intestinal absorption, metabolism and cytotoxicity [30], [31], [32], [33], [34], [35]. A previous study reported that chromium (unspecified valence) increased lipid peroxidation in Caco-2 cells [36]. However, to our knowledge, the current study is the first to explore the genotoxicity of Cr(VI) in undifferentiated and differentiated Caco-2, a cell line highly relevant to the intestinal carcinogenicity of ingested Cr(VI).

Herein, we assess DNA damage in differentiated and undifferentiated Caco-2 cells following exposure to Cr(VI) using high content analysis, which is an imaging based multi-parametric approach to cell analysis at the single-cell level. Advantages of this method include: fully automated and unbiased image analysis, simultaneous analysis of multiple parameters in the same cell population, and high throughput capability [37], [38]. Following treatment of Caco-2 with Cr(VI), DNA damage was monitored by the presence of phosphorylated histone variant H2AX (γ-H2AX) and 8-hydroxydeoxyguanosine (8-OHdG) in the nucleus. 8-Hydroxydeoxyguanosine is a marker of oxidative DNA damage, whereas H2AX phosphorylation is a sensitive indicator of DNA double strand breaks (DSB) and other forms of DNA damage that can arise from direct interaction between a chemical and DNA, ROS, replication stress, and DNA misrepair [39], [40]. Additionally, MN assays were conducted in Caco-2, CHO-K1, and A549 cell lines. Together, these studies can help inform the *in vivo* toxicity and carcinogenicity of Cr(VI) in the small intestine.

## Results

### Cytotoxicity in Undifferentiated and Differentiated Caco-2

**Figure 1:**
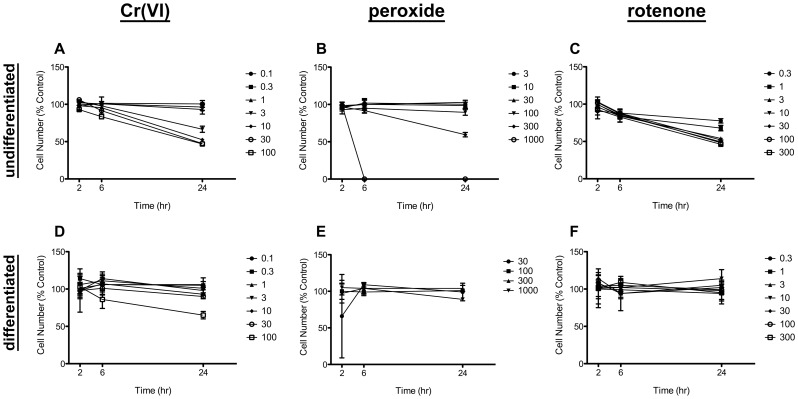
Cell viability in undifferentiated and differentiated Caco-2. Undifferentiated (A–C) and differentiated (D–F) cells were treated with the indicated concentrations (µM) of Cr(VI) (A, D), hydrogen peroxide (B, E) or rotenone (C, F). Cell numbers were measured after 2, 6, and 24 hours of incubation. Data shown represents two independent experiments each in triplicate. Data are expressed as % Control of vehicle-treated (water for hydrogen peroxide and Cr(VI), DMSO for rotenone) cells at same time point. Data are plotted as mean ±s.d.

Cytotoxicity was assessed using Hoechst stain to identify cell number as well as determine nuclear size. Undifferentiated and differentiated Caco-2 cells were treated with Cr(VI) and two compounds known to alter cellular redox status and induce oxidative DNA damage, *viz*. hydrogen peroxide and rotenone. Treatment of undifferentiated Caco-2 cells with these compounds resulted in a dose-dependent reduction in cell numbers at 24 hr post-treatment (**Fig. 1A–C**). In contrast, cell numbers of differentiated Caco-2 cells were less affected by treatment with the three compounds (**Fig. 1D–F**), indicating that differentiated cells were more resistant to chemical-induced cytotoxicity; only 100 µM Cr(VI) reduced cell number.

**Figure 2:**
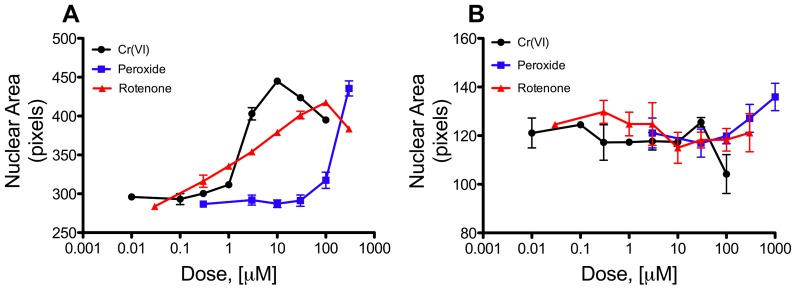
Nuclear morphology in undifferentiated and differentiated Caco-2. Nuclear area in undifferentiated (A) and differentiated (B) Caco-2. Nuclear area was measured in same cells as in Figure 1. Data shown represents two independent experiments, each in triplicate. Data are plotted as mean ± SEM of nuclear area in actual pixels. Control values are plotted at 10-fold below the lowest treatment dose.

Nuclear area (or size) provides additional information on the health of cells. Increased nuclear area is often observed in compounds that block cell cycle and/or induce DNA damage [41], [42]. Changes in nuclear area in differentiated and undifferentiated Caco-2 cells at 24-hr (**Fig. 2**) were in close agreement with changes in cell number at 24-hr (**Fig. 1**). In the undifferentiated Caco-2, nuclear area was significantly increased at ≥3 µM Cr(VI), ≥100 µM peroxide, and in all rotenone concentrations (p<0.05 by ANOVA followed by Dunnett’s test). In differentiated Caco-2, nuclear area was significantly decreased at 100 µM Cr(VI) (**Fig. 2B**). The nuclear area of differentiated Caco-2 were smaller due, in part, to the difference in cell shape. Undifferentiated Caco-2 cells were flat and circular making nuclear measurements easier, whereas differentiated Caco-2 cells were columnar in shape and thus more difficult to measure the nucleus accurately. Nevertheless, differentiated cells treated with 100 µM Cr(VI) exhibited decreased nuclear size (**Fig. 2B**) and increased nuclear staining intensity (data not shown) compared to untreated cells. These findings suggest that the reduction in cell numbers in differentiated cells (**Fig. 1D**) might have been due to apoptosis. However, typical apoptosis markers p53 and annexin-V were not elevated as a result of Cr(VI) exposure (see below). Taken together, data in **Figs. 1** **and** **2** indicate that differentiated intestinal cells were resistant to Cr(VI)-induced cytotoxicity, and that treatment with ≤1 µM Cr(VI) did not induce obvious signs of cytotoxicity or cell cycle arrest in undifferentiated Caco-2 cells.

### Nuclear Staining of γ-H2AX and 8-OHdG in Undifferentiated Caco-2 Cells

**Figure 3:**
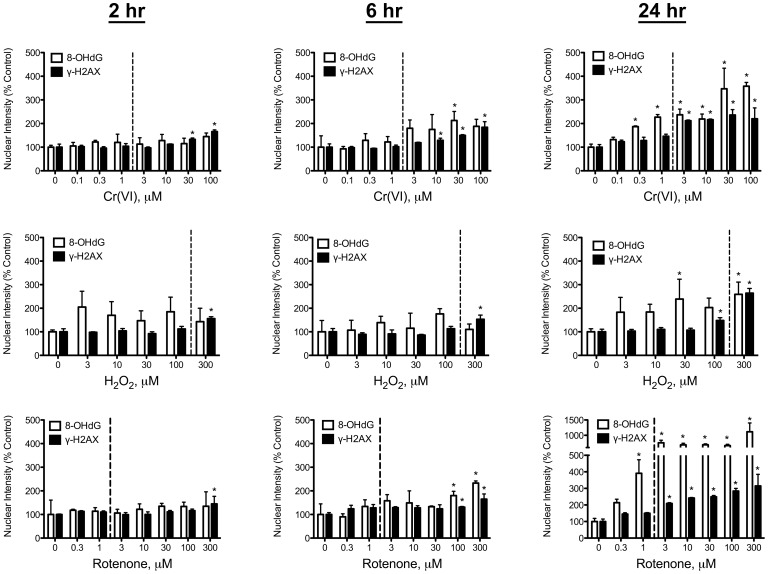
DNA damage in undifferentiated Caco-2. Nuclear staining intensity of 8-OHdG and γ-H2AX in undifferentiated Caco-2 cells. Cells were seeded at density 1×10^4^ cells/100 µl/well, grown for 24 hr, then treated with the indicated concentrations of Cr(VI) (top), hydrogen peroxide (middle) or rotenone (bottom). Nuclear staining intensity was measured after 2, 6, and 24 hours of incubation. Concentrations to the right of the dotted line of each plot were cytotoxic (reduced cell numbers) at 24 hr of exposure. Data are expressed as % Control, and represent mean ±s.d. (n = 3, where n is number of individual replicates). ^*^p<0.05 by ANOVA followed by Dunnett’s test.

DNA damage in the form of 8-OHdG formation and H2AX phosphorylation were measured in Caco-2 cells at 2, 6 and 24 hrs after treatment. Plots of nuclear staining intensity in undifferentiated Caco-2 cells are shown in **Fig. 3** (concentrations to the right of the dashed lines are cytotoxic at 24 hr). At 2 hours post exposure, all three compounds increased γ-H2AX staining intensity at the highest concentrations without apparent increase in 8-OHdG staining intensity. By 6 hours, 8-OHdG staining in peroxide treated cells was elevated, although the data were not statistically significant. In contrast, γ-H2AX staining was significantly increased at 300 µM peroxide. Rotenone treatment for 6 hours resulted in significant increases in both 8-OHdG and γ-H2AX at 100 and 300 µM. Treatment with Cr(VI) for 6 hours increased 8-OHdG fluorescence, but only significantly at 30 µM. In contrast, γ-H2AX staining was significantly increased at ≥10 µM. Notably, there were no significant increases in 8-OHdG or γ-H2AX at non-cytotoxic concentrations for any of the compounds at 2–6 hours post exposure.

**Figure 4:**
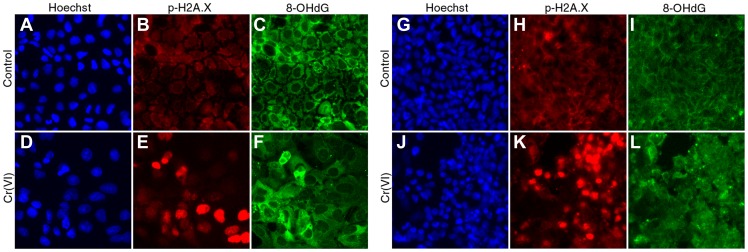
Representative images of 8-OHdG and γ-H2AX fluorescence in Caco-2. Representative images of 8-OHdG and γ-H2AX staining in control and 100 µM Cr(VI) treated undifferentiated (A–F) and differentiated (G–L) Caco-2 cells at 24 hr. All images were taken with the same magnification (20X). Intensity of 8-OHdG and γ-H2AX immunostaining was determined in nuclear area identified by staining with Hoechst 33342.

After 24-hour treatment at non-cytotoxic concentrations, all 3 compounds increased 8-OHdG staining. Only peroxide increased γ-H2AX staining at the highest non-cytotoxic concentration (100 µM). In contrast, Cr(VI) and rotenone increased 8-OHdG staining without concomitant increases in γ-H2AX staining at non-cytotoxic concentrations. At cytotoxic concentrations, all 3 compounds significantly increased both 8-OHdG and γ-H2AX nuclear staining intensity. **Figure 4A–F** shows representative images of 8-OHdG and γ-H2AX staining in control and 100 µM Cr(VI) treated undifferentiated Caco-2 cells at 24 hr. As noted in other studies, 8-OHdG staining also occurs outside the nucleus due to the presence of RNA and mitochondrial DNA.

### Comparison of EC_50_ Values for γ-H2AX and 8-OHdG in Undifferentiated Caco-2 Cells

The half maximal effective concentration (EC_50_) is the concentration of a toxicant which induces a response halfway between the base level and maximum after the exposure time. It is a commonly used measure of toxicant’s potency. To compare the relative potency of the three compounds for 8-OHdG and γ-H2AX nuclear staining, EC_50_ values were derived from dose-response modeling. For Cr(VI) and peroxide, the EC_50_ values for 8-OHdG were lower than γ-H2AX (**Table 1**; **Fig. S1**). Overall, these data indicate that Cr(VI), peroxide and rotenone increase oxidative DNA damage at lower concentrations than H2AX phosphorylation under longer term exposure (i.e. 24 hr), and that γ-H2AX formation was correlated with cytotoxicity – especially for Cr(VI) and rotenone.

**Table 1:** EC_50_ values for 8-OHdG and γ-H2AX Nuclear Staining in Undifferentiated Caco-2.

	8-OHdG (µM)	γ-H2AX (µM)
Cr(VI)	0.21	0.88
Rotenone	0.99	1.1
Peroxide	5.5	1.16

### Nuclear Staining of γ-H2AX and 8-OHdG in Differentiated Caco-2 Cells

**Figure 5:**
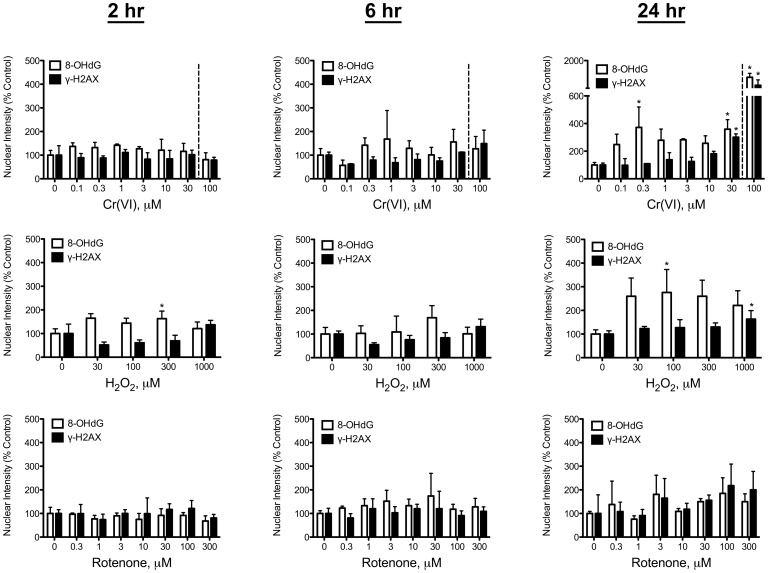
DNA damage in differentiated Caco-2. Nuclear staining intensity of 8-OHdG and γ-H2AX in differentiated Caco-2 cells. Cells were seeded at density 2×10^4^ cells/100 µl/well in Collagen I coated plates, grown for 21 days, then treated with the indicated concentrations of Cr(VI) (top), hydrogen peroxide (middle) or rotenone (bottom). Nuclear staining intensity was measured after 2, 6, and 24 hours of incubation. Concentrations to the right of the dotted line of each plot were cytotoxic (reduced cell numbers) at 24 hr of exposure. Data are expressed as % Control, and represent mean ±s.d. (n = 3, where n is number of individual replicates). ^*^p<0.05 by ANOVA followed by Dunnett’s test.

With the exception of 300 µM peroxide, the 3 chemicals did not significantly alter 8-OHdG or γ-H2AX staining in differentiated Caco-2 cells after 2 to 6 hrs of exposure (**Fig. 5**). After 24-hr treatment, peroxide increased 8-OHdG, albeit only significantly at 100 µM; in contrast, γ-H2AX staining was increased at 1 mM. Rotenone did not significantly increase 8-OHdG or γ-H2AX staining at any treatment concentration. Exposure to Cr(VI) for 24 hours elicited increases in 8-OHdG at all concentrations, albeit only significantly at 0.3, 30 and 100 µM (**Fig. 5**). Staining of γ-H2AX was increased at 30 and 100 µM, although the latter concentration was cytotoxic (see **Fig. 1D**). In contrast to undifferentiated Caco-2 cells treated with Cr(VI), 8-OHdG and γ-H2AX staining was more closely correlated in differentiated cells treated with Cr(VI). **Fig. 4G–L** shows representative images of 8-OHdG and γ-H2AX staining in control and 100 µM Cr(VI) treated differentiated Caco-2 cells. This figure also shows the difference in cell density between proliferating and differentiated cells (**Fig. 4A&G**), as well as disruption of the monolayer in differentiated Caco-2 cells treated with 100 µM Cr(VI) for 24 hr (**Fig. 4G&J**). Overall, these data indicate that differentiated Caco-2 are less sensitive to Cr(VI) than undifferentiated Caco-2.

### Further Evaluation of Effects in Differentiated Caco-2 Cells

*In vivo* studies indicate that damage to the intestinal villus is one of the earliest and most sensitive effects of Cr(VI) exposure [2], [20]. In mice, Cr(VI) exposure results in changes in redox (*viz*. GSH/GSSG ratio), villus cytoplasmic vacuolization, and karyorrhectic nuclei in the villus – whereas the intestinal crypts appear only to undergo proliferation in response to villus cytotoxicity [20]. To explore the effects of Cr(VI) on villus, we further stained differentiated Caco-2 cells with markers of apoptosis (p53 and annexin-V), autophagy (LC3B), and endoplasmic reticulum (ER) stress (ATF6). The latter was performed because toxicogenomic analyses indicated ER stress responses in the duodenum following Cr(VI) exposure [43].

**Figure 6:**
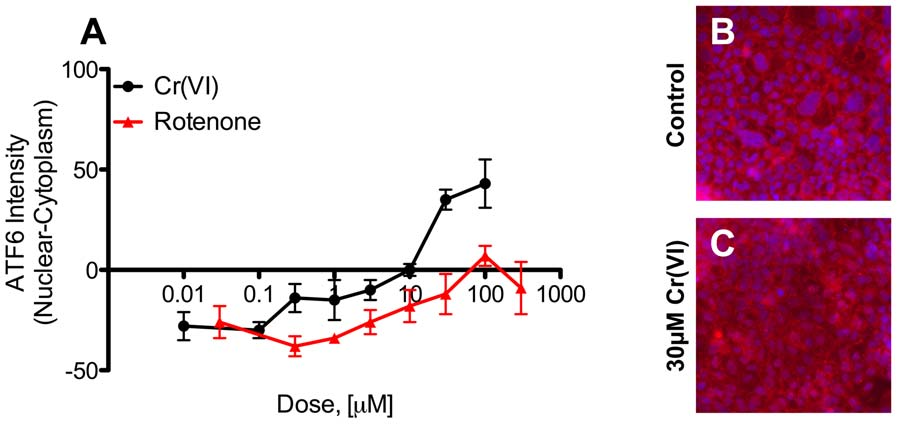
ATF6 localization in differentiated Caco-2. (A) Difference between nuclear and cytoplasmic expression of ATF6. Positive slope indicates translocation of ATF6 into the nucleus. (B) Dual channel overlay of Hoechst (blue) and ATF6 (red) fluorescence in untreated Caco-2 cells. Blue nuclei indicate relative absence of ATF6 in nucleus. (C) Dual channel overlay of Hoechst and ATF6 fluorescence in Caco-2 treated with 30 µM Cr(VI) for 24 hr. Loss of blue nuclei indicate movement of ATF6 into the nucleus. Data are plotted as mean ±s.d. (n = 3, where n is number of individual replicas).

Staining of differentiated Caco-2 cells with p53 and annexin-V resulted in no apparent changes in immunofluorescence at 24 hr by any of the three compounds (data not shown). Similarly, there was no change in LCB3, which is normally present on autophagic vesicles. In contrast, there was a dose-dependent change in ATF6, a transcription factor that regulates the unfolded protein response (UPR). In unstressed conditions, ATF6 resides in the ER, but ER stress results in cleavage of ATF6 and translocation into the nucleus. As shown in **Fig. 6A**, Cr(VI) (and to a lesser extent rotenone) caused a dose dependent translocation of ATF6 from the cytoplasm to the nucleus. In untreated cells, ATF6 is located outside the nucleus and thus the mathematical difference in nuclear and cytoplasmic fluorescence is negative (**Fig. 6A**). This is evident in **Fig. 6B** where Hoechst nuclear fluorescence can be readily seen as blue staining, and ATF6 as red cytoplasmic staining. Treatment with Cr(VI) results in a decrease in cytoplasmic ATF6 fluorescence and an increase in nuclear ATF6 fluorescence, resulting in a positive value along the y-axis at higher Cr(VI) concentrations (**Fig. 6A**). This can be seen in **Fig. 6C** where the nuclear Hoechst fluorescence is obscured by ATF6 nuclear fluorescence. Notably, there was significant elevation in 8-OHdG and γ-H2AX staining (**Fig. 5**) at concentrations where ATF6 was present in the nucleus. Together, these data suggest that Cr(VI) may induce an UPR in differentiated intestinal cells that is, in part, due to oxidative stress.

### Micronucleus Formation in CHO-K1 and A549 Cells

For continuity with the previous assays, MN formation was assessed in Caco-2 cells; however, the background MN levels were not ideal, and it is recommended that only cells with very low background MN levels be used for testing [44]. Therefore, two alternative cells models were selected. CHO-K1 cells were selected because they are a well-accepted *in vitro* cell model that is recommended by the Organisation for Economic Co-operation and Development (OECD) for assessing genotoxic potential via the MN assay [44]. Exposure to Cr(VI) reduced viability by ∼50% or more at ≥32 µM (**Table 2**). The percentage of bi-nucleated cells was significantly reduced at ≥32 µM Cr(VI) – suggesting treatment-induced cell cycle arrest at ≥32 µM. At these cytotoxic and/or cytostatic concentrations Cr(VI) increased MN frequency (**Table 2**). In contrast, the clastogenic positive control MMC significantly (p<0.001) increased MN frequency at concentrations that did not greatly reduce cell numbers or the percentage of bi-nucleated cells (**Table 2**).

**Table 2:** Micronuclei Formation in CHO-K1 Cells.

	Cell Count (%Control)			% Binucleated			% Micronuclei^a^		
Control	99.94	±	6.66	68.99	±	2.58	1.41	±	0.68
MMC (µM)									
1	98.56	±	6.39	70.22	±	1.56	1.69	±	0.90
10	101.70	±	5.81	69.33	±	2.40	1.86	±	0.90
100	91.11	±	7.46^b^	62.33	±	1.94^b^	4.14	±	1.85^b^
Cr(VI) (µM)									
0.1	99.67	±	4.56	71.44	±	1.24	1.32	±	0.80
0.3	111.60	±	12.64	71.11	±	2.15	1.51	±	0.77
1	108.00	±	10.55	70.22	±	2.22	1.71	±	0.86
3.2	98.89	±	7.25	69.44	±	1.94	1.79	±	1.06
10	81.33	±	4.27^b^	62.78	±	2.17	1.70	±	1.15
32	55.11	±	5.04^b^	12.00	±	1.00^b^	3.96	±	4.03
100	19.89	±	1.27^b^	15.22	±	1.39^b^	5.86	±	7.16

a % micronuclei in binucleated cells.

b statistically significant by ANOVA followed by Dunn’s *post hoc* test (p<0.05).

data are mean ±SD.

In addition to CHO-K1 cells, the human lung adenocarcinoma epithelial A549 cell line was assessed for MN formation because Cr(VI) inhalation exposure is associated with increased risk of lung cancer. Treatment of A549 cells with Cr(VI) caused statistically significant (p<0.001) increases in cytotoxicity and decreases in the percentage of bi-nucleated cells at ≥10 µM (**Table 3**). Cr(VI) also caused relatively small but statistically significant (p<0.05) increases in cytotoxicity and decreases in the percentage of bi-nucleated cells at 3.2 µM. At this concentration, the frequency of MN in bi-nucleated cells was slightly (but statistically significant; p<0.05) increased from 1.47±0.50 to 2.12±0.41% (**Table 3**). At higher concentrations the A549 cells were essentially dead. MMC significantly increased (p<0.001) MN frequency from 1.47±0.50 to 6.89±2.24% (**Table 3**). To explore whether this increase in MN frequency arose from clastogenic or aneugenic mechanisms, cells were stained with fluorescent antibodies for the kinetochore protein centromere protein-B (CENP-B). Treatment of A549 cells with MMC resulted in increases in kinetochore-negative MN (consistent with the clastogenic properties of MMC), whereas treatment with Cr(VI) did not alter the percentage of kinetochore negative MN (data not shown).

**Table 3:** Micronuclei Formation in A549 Cells.

	Cell Count (%Control)			% Binucleated			% Micronuclei^a^		
Control	99.94	±	8.113	59.62	±	5.78	1.47	±	0.50
MMC (µM)									
1	98.78	±	3.80	58.78	±	3.35	1.41	±	0.37
10	95.22	±	3.31	57.00	±	2.78	1.70	±	0.27
100	78.78	±	5.52^b^	46.78	±	3.07^b^	6.89	±	2.24^b^
Cr(VI) (µM)									
0.1	98.44	±	5.62	58.56	±	2.35	1.51	±	0.35
0.3	94.33	±	5.43	56.00	±	1.94	1.62	±	0.45
1	93.11	±	5.80	55.33	±	2.40	1.46	±	0.33
3.2	87.00	±	4.30^b^	51.67	±	1.80^b^	2.12	±	0.41^c^
10	33.33	±	2.12^b^	20.00	±	1.32^b^	2.12	±	0.81^c^
32	19.22	±	2.11^b^	11.33	±	1.23^b^	0.00	±	0.00^b^
100	25.78	±	1.99^b^	15.33	±	1.41^b^	0.00	±	0.00^b^

a % micronuclei in binucleated cells.

b statistically significant by ANOVA followed by Dunn’s *post hoc* test (p<0.05).

c statistically significant by ANOVA followed by Dunnett’s *post hoc* test (p<0.05).

data are mean ±SD.

## Discussion

The *in vitro* MN assay is a well-accepted test for assessing the genotoxic potential of a compound [44]. We employed CHO-K1 cells and HCA because the former is recommended by the OECD for *in vitro* genotoxicity testing [44], and because the latter allows for efficient screening of multiple fields of cells (∼9000 bi-nucleated cells per treatment group) in an unbiased objective manner [45]. Cr(VI) did not significantly increase MN in CHO-K1, except at concentrations that decreased cell number and mitosis (bi-nucleated cells). Given these negative findings, we explored whether Cr(VI) could induce MN in A549 cells, which are of potential relevance due to associations between inhaled Cr(VI) exposure and lung cancer. In these cells, Cr(VI) increased MN frequency slightly, but again only at concentrations that also decreased the number of bi-nucleated cells. Together, the findings from these two cell lines suggest that Cr(VI) is only weakly genotoxic.

Other studies have reported increases in MN frequency in cells that are first pretreated with mM concentrations of dehydroascorbic acid (DHA) in order to elevate cellular ascorbate to levels reported in tissues and freshly isolated cells [46], [47]. However, ascorbate is known to interact with culture media constituents and generate peroxide, which can potentiate the genotoxicity of other compounds [48], [49], [50], [51], [52]. It is also known that DHA reduction to ascorbate is mediated nonenzymatically by GSH as well as enzymatically through GSH-dependent and NADPH-dependent reactions [53], [54]; thus loading cells with ascorbate may alter cellular redox and, contrary to some arguments [46], may not accurately recapitulate *in vivo* tissue conditions.

Despite the weak evidence for genotoxicity in the MN assay, Cr(VI) is well documented to induce DNA lesions, including Cr-DNA adducts, DNA-protein crosslinks, DNA-Cr-DNA crosslinks, and oxidative DNA damage (see recent reviews on Cr(VI) carcinogenicity [8], [9], [10], [12]). Because chronic ingestion of Cr(VI) has been shown to cause intestinal tumors in the mouse small intestine [2], Caco-2 cells were used as an *in vitro* intestinal model to study the potential for Cr(VI) to induce DNA damage in the small intestine. In short-term culture, Caco-2 cells are undifferentiated and proliferate, and thus share some characteristics with intestinal crypt enterocytes. When grown for ∼21 days, Caco-2 differentiate and develop morphological characteristics of mature villus enterocytes. Thus, undifferentiated and differentiated Caco-2 cells recapitulate intestinal enterocytes along the crypt-villus axis, respectively.

A major finding from this study was that differentiated Caco-2 cells were more resistant to Cr(VI), peroxide, and rotenone than undifferentiated/proliferating Caco-2 cells. Neither rotenone nor peroxide induced cytotoxicity in differentiated cells, and the cytotoxicity of Cr(VI) was greatly diminished. In contrast, all three compounds reduced the number of undifferentiated Caco-2 cells in a concentration and time-dependent manner. Cr(VI) treatment increased both 8-OHdG and γ-H2AX nuclear staining at concentrations that reduced cell numbers and increased nuclear size, suggesting that DNA damage was associated with cytotoxicity and/or cell cycle arrest. Findings with respect to greater H2AX phosphorylation in proliferating but not differentiated Caco-2 cells treated with Cr(VI) are consistent with previous reports indicating that Cr(VI) primarily induced γ-H2AX nuclear staining in normal human fibroblasts in S-phase, in part due to replication stress [55]. Although GSH levels may be higher in undifferentiated than differentiated Caco-2 cells [56], oxidative DNA damage appeared to be greater in undifferentiated cells. Similar patterns of differentiation-dependent disparities in oxidative DNA damage in response to oxidants have been observed in other cells types [57]. Notably, cellular protein content is several-fold higher in differentiated than undifferentiated Caco-2 cells [56], and thus increases in antioxidant enzymes and DNA repair enzymes might partially explain the recalcitrance of differentiated Caco-2 cells. However, it is also conceivable that the higher GSH in undifferentiated Caco-2, by reducing Cr(VI) to Cr(III) and thereby generating reactive intermediates, paradoxically potentiated oxidative DNA damage in these cells.

Another major finding from this Caco-2 model is that Cr(VI) increased 8-OHdG staining at lower concentrations than γ-H2AX staining as evidenced by their respective EC_50_ values. The EC_50_ for 8-OHdG staining was approximately 4-fold lower than for γ-H2AX staining (0.21 vs. 0.88 µM). The mechanism of Cr(VI)-induced 8-OHdG formation is not clear. Previous studies have shown that Cr(VI)-induced toxicity in A549 cells could be ameliorated by catalase, suggesting involvement of peroxide formation [58]. Although peroxide treatment was far less potent than Cr(VI) in inducing 8-OHdG nuclear staining, Cr(VI) reduction involves binding to antioxidants such as GSH, formation of unstable reactive chromium intermediates such as Cr(V), and generation of ROS (e.g. peroxide) – and is therefore likely to affect cells differently than peroxide alone. Interestingly, several studies have reported that continuous passage (4–24 weeks) of human bronchial epithelial Beas-2B cells in 0.25–5 µM Cr(VI) can cause transformation [13], [14], [15]. Based on data herein, 0.25–5 µM Cr(VI) may be high enough to increase 8-OHdG formation that could eventually lead to DNA damage, mutation and transformation. Indeed, Wang et al. (2011) showed that 2 µM Cr(VI) increased ROS in Beas-2B cells, and that transformation of Beas-2B cells during chronic exposure to 0.25 µM Cr(VI) was ameliorated by transfection of plasmids containing superoxide dismutase, catalase, or RNA inhibitors of NADPH oxidase (NOX) [15]. Certain NOX family members and the related dual oxidases are highly expressed in Caco-2 cells and throughout the gastrointestinal tract [59], [60], and their propensity to generate superoxide might play a role in Cr(VI) cytotoxicity and carcinogenicity in the small intestine. Notably, macrophages express NOX enzymes and Cr(VI) exposure was accompanied by infiltration of macrophages into the intestine [2], [17], [20], [61].

H2AX phosphorylation is a sensitive indicator of DNA DSB, which can results from chemical-induced DNA damage or damage introduced as a result of DNA repair [39], [40]. It is suggested that Cr-DNA binary adducts may represent more than 75% of all Cr-DNA adducts (binary and ternary combined), and that these binary adducts have relatively weak mutagenic potential [12]. Repair of these adducts is mediated primarily by nucleotide excision repair (NER) pathways [12], but it has been shown that the number of mutations in Cr(VI)-treated cells is lower in cells deficient in NER as well as base excision repair (BER) [62] – suggesting that much of Cr(VI)-induced DNA mutations occur as a result of DNA misrepair. Ternary Cr-DNA adducts (e.g. GSH-Cr-DNA) are less common but may be more mutagenic, as these lesions can lead to DNA DSB formation and replication inhibition/stress [10], [11], [12], [55]. Replication inhibition induces mismatch repair (MMR) processes that can introduce DNA DSB and H2AX phosphorylation [12], [39], [55], [63]. Thus, the repair of Cr-DNA adducts by NER, BER and MMR can all result in DNA DSB and H2AX phosphorylation [39]. In addition to Cr-DNA adducts, ROS from Cr(VI) reduction to intermediate valences as well as changes in cellular redox status can generate single and double strand breaks that result in H2AX phosphorylation [39], [40], [64]. Clearly then, the Cr(VI)-induced H2AX phosphorylation described herein cannot be attributed to any single type of lesion. Importantly, it has been suggested that H2AX phosphorylation serves as a general indicator of cellular stress, DNA damage, and genomic integrity [39]; thus the absence of γ-H2AX staining below 3 µM Cr(VI) may suggest that the 8-OHdG is not indicative of severe DNA damage. In support of this, increases in γ-H2AX at ≥3 µM Cr(VI) corresponded to increases in nuclear area – which may be indicative of cell cycle arrest due to the presence of DNA DSB. Although we cannot rule out the possibility that low levels of adducts or damage occurred and were either efficiently repaired or insufficient to increase γ-H2AX staining, phosphorylation of H2AX is reported to be orders of magnitude more sensitive than other methods of DNA DSB detection [39]. Nevertheless, future analyses using additional markers of DNA damage (e.g. TUNEL staining or Comet assay) as well as assessment of Cr-DNA binding could further inform the genotoxicity of Cr(VI) in Caco-2.

The Caco-2 model described herein may provide additional insight into *in vivo* mechanistic studies recently published on the effects of Cr(VI) on the rodent small intestine [20], [26], [27]. In these studies, mice were exposed for 90 days to 0.1–182 mg/L Cr(VI), or approximately 2–3,000 µM Cr(VI) [20]. Although Cr(VI) is reduced to Cr(III) in gastric fluid [65], it is likely that much of the Cr(VI) is not reduced at the higher treatment concentrations as evidenced by tissue chromium levels [20]. Despite the presence of Cr(VI) in the lumen, toxicity was confined to the villus, which was accompanied by hyperplasia in the crypt [20]. Analysis of duodenal crypts indicated no increases in apoptotic index or aberrant nuclei (e.g. MN) after 7 or 90 days of exposure to Cr(VI) in drinking water [26]. Moreover, changes in *K-ras* mutation frequency, an early mutation often found in intestinal adenomas [66], were not detected in scraped duodenal mucosa cells (including crypts) after 90 days of exposure [27]. In contrast to these *in vivo* data indicating an apparent absence of toxicity in intestinal crypts, undifferentiated/proliferating Caco-2 cells were far more sensitive to Cr(VI)-induced toxicity than differentiated Caco-2, and exhibited no Cr(VI)-induced increase in proliferation. These disparate responses between undifferentiated Caco-2 and intestinal crypt cells suggest that the latter were not directly exposed to Cr(VI) *in vivo*, and that crypt hyperplasia was likely due to toxicity in the villus. However, it cannot be ruled out that oxidative species either from the lumen or surrounding cells (e.g. macrophages), together with proliferative pressure, contributed to intestinal carcinogenesis in mice. A similar villus injury/crypt hyperplasia mechanism has been proposed for the pesticides captan and folpet – which, like Cr(VI), react with thiols, induce duodenal villus toxicity, crypt hyperplasia and intestinal tumors in mice but not rats [67], [68].

The affect of Cr(VI) on ATF6 translocation to the nucleus in differentiated Caco-2 cells is consistent with toxicogenomic responses following Cr(VI) exposure, *viz*. activation of ER stress transcription factors ATF4 and XBP1 in the duodenum [43]. ATF6, ATF4, and XBP1 are involved in ER stress response, and ATF4 plays an important role in the regulation of autophagy, which is characterized by autophagosomal vacuoles in the cytoplasm [69]. These *in vitro* and *in vivo* findings further suggest that the toxicity in villi is related to oxidative stress. The lack of Cr(VI)-induced activity of p53 and Annexin V in differentiated Caco-2 cells is consistent with the absence of apoptosis in villus enterocytes (although Cr(VI) did induce apoptosis in other cells of the villous lamina propria) [20].

In summary, proliferating/undifferentiated Caco-2 cells were more sensitive to Cr(VI) than differentiated Caco-2 cells, and oxidative DNA damage was detected at lower concentrations than H2AX phosphorylation. The findings suggest that *in vitro* studies showing the transformation of cells following long-term culture with low µM Cr(VI) concentrations may be due to prolonged increases in oxidative stress. The findings also suggest that *in vivo* studies indicating villus but not crypt toxicity following ingestion of Cr(VI) in drinking water imply that crypt cells were not in direct contact with Cr(VI). Additional investigations are underway to further explore toxicity and carcinogenicity of Cr(VI) in the small intestine.

## Materials and Methods

### Cell Culture

Caco-2 cells (ATCC HTB-37) were maintained and seeded in RPMI-1640 medium (Thermo Fisher Scientific) containing 20% FBS (Thermo Fisher Scientific), 2 mM L-Glutamine (Thermo Fisher Scientific), 1 mM sodium pyruvate (Thermo Fisher Scientific), 1X non-essential amino acids (Thermo Fisher Scientific), 100 U/ml penicillin and 100 µg/ml streptomycin (Thermo Fisher Scientific). CHO-K1 cells (ATCC, CCL-61) were maintained and seeded in F12-K medium (Thermo Fisher Scientific) containing 10% FBS (Thermo Fisher Scientific), 100 U/ml penicillin and 100 µg/ml streptomycin (Thermo Fisher Scientific). A549 cells (ATCC, CCL-185) were maintained and seeded in F12-K medium (Thermo Fisher Scientific) containing 10% FBS (Thermo Fisher Scientific), 2 mM L-Glutamine (Thermo Fisher Scientific), 1 mM sodium pyruvate (Thermo Fisher Scientific), 100 U/ml penicillin and 100 µg/ml streptomycin (Thermo Fisher Scientific).

For assays in undifferentiated/proliferating Caco-2 cells, cells were seeded at a density of 1×10^4^ cells/100 µl/well in Nunc Edge 96-well microplates (Thermo Fisher Scientific) 24 hr prior to incubation with compounds. For assays in differentiated Caco-2 monolayers, cells were seeded at a density of 2×10^4^ cells/100 µl/well in Collagen I (BD Biosciences) coated Nunc Edge 96-well microplates (Thermo Fisher Scientific), and cultured for 21 days prior to incubation with compounds, with a media change every other day. Test compounds include: Cr(VI) in the form of sodium dichromate dihydrate (SDD) (0.1–100 µM), hydrogen peroxide (3–1000 µM), and rotenone (0.3–300 µM) (all from Sigma). Stock solutions of SDD and hydrogen peroxide were prepared in H_2_O. Rotenone was prepared in DMSO at a final concentration of 0.25% DMSO. Cells were incubated with all compounds for 2 hr, 6 hr, and 24 hr.

### Immunocytochemistry

After incubation cells were fixed with 4% paraformaldehyde in PBS (EMS). Harvested cells were incubated with 0.05 M NaOH in 40% ethanol for 12 min and 250 µg/ml RNAse A for 60 min at 37°C as previously described [70]. For immunocytochemistry, plates were incubated in 1X blocking buffer (Thermo Fisher Scientific) for one hour prior to addition of primary and secondary antibody (each incubated for 1 hr at room temperature). Primary antibodies: mouse monoclonal 8-Hydroxyguanosine antibody (Abcam ab62623), rabbit polyclonal gamma H2A.X (phospho S139) antibody (Abcam ab2893), mouse monoclonal p53(Abcam ab1101), mouse monoclonal ATF6 (Abcam ab11909), rabbit polyclonal LC3B (Abcam ab63817), rabbit polyclonal Annexin V (Abcam ab14196). Secondary antibodies: Alexa Fluor 488 goat anti-mouse IgG (H+L) (Invitrogen A-11001), Alexa Fluor 647 goat anti-rabbit IgG (H+L) (Invitrogen A-21244). Hoechst 33342 was used as a nuclear counterstain for automated cell identification. This counterstain was also used to determine nuclear size (image area) and nuclear staining intensity. Plates were imaged using the Thermo Scientific ArrayScan VTI HCS Reader (Thermo Fisher Scientific) and analyzed using the Compartmental Analysis BioApplication (Thermo Fisher Scientific). Immunostaining-based parameters and nuclear staining based parameters were determined and analyzed in the same image set for each field. No less then 3 fields (>500 cells) where analyzed for each data replicate.

### Micronucleus Assay

The CHO-K1 and A549 micronucleus assays were performed according to the Cellomics Micronucleus Kit (Thermo Fisher Scientific) instructions. CHO-K1 cells were seeded at a density of 3×10^3^ cells per 100 µl/well 18 hrs prior to chemical treatment. A549 cells were seeded at 5×10^3^ cells per 100 µl/well 18 hrs prior to chemical treatment. Both CHO-K1 and A549 cells were treated for 20 hr with Cr(VI) in the form of SDD (Sigma), and mitomycin C (MMC; Tocris). Cells were treated with 6 µg/ml Cytochalasin B following compound treatment and incubated for a further 27.5 hours. Micronucleus assay in Caco-2 cells was performed as previously described [71]. The fraction of MN with centromeric chromatin was determined in A549 cells by immunofluorescent staining using anti-kinetochore antibodies targeting CENPB (Abcam ab25734). Cells were co-stained with Hoechst 33342 and SYTO Red (Life Technologies) nucleic acid stains to reveal nuclei, micronuclei and cell bodies. For all cell lines, plates were imaged using ArrayScan VTI (Thermo Fisher Scientific). No less than one thousand cells or 40 fields (20X) were acquired and analyzed for each well. All images were analyzed using the Micronucleus BioApplication (Thermo Fisher Scientific).

### Statistics

All data were analyzed by one-way ANOVA followed by Dunnett’s or Dunn’s *post hoc* tests using Prism 5 for Mac OS X (GraphPad Software, Inc.). For EC_50_ derivation, data were normalized and modeled using nonlinear regression in Prism 5 for Mac OS X.

## Supporting Information

Figure S1 Modeling of nuclear staining intensity in proliferating Caco-2 cells at 24 hr. Concentrations shown include up to first two toxic concentrations.
(TIF)
