# Supplementary figures and images for: Assessment of Cr(VI)-Induced Cytotoxicity and Genotoxicity Using High Content Analysis
Source: PLoS One. 2012 Aug 8;7(8):e42720. doi: 10.1371/journal.pone.0042720 (PMC3414448; doi:10.1371/journal.pone.0042720)

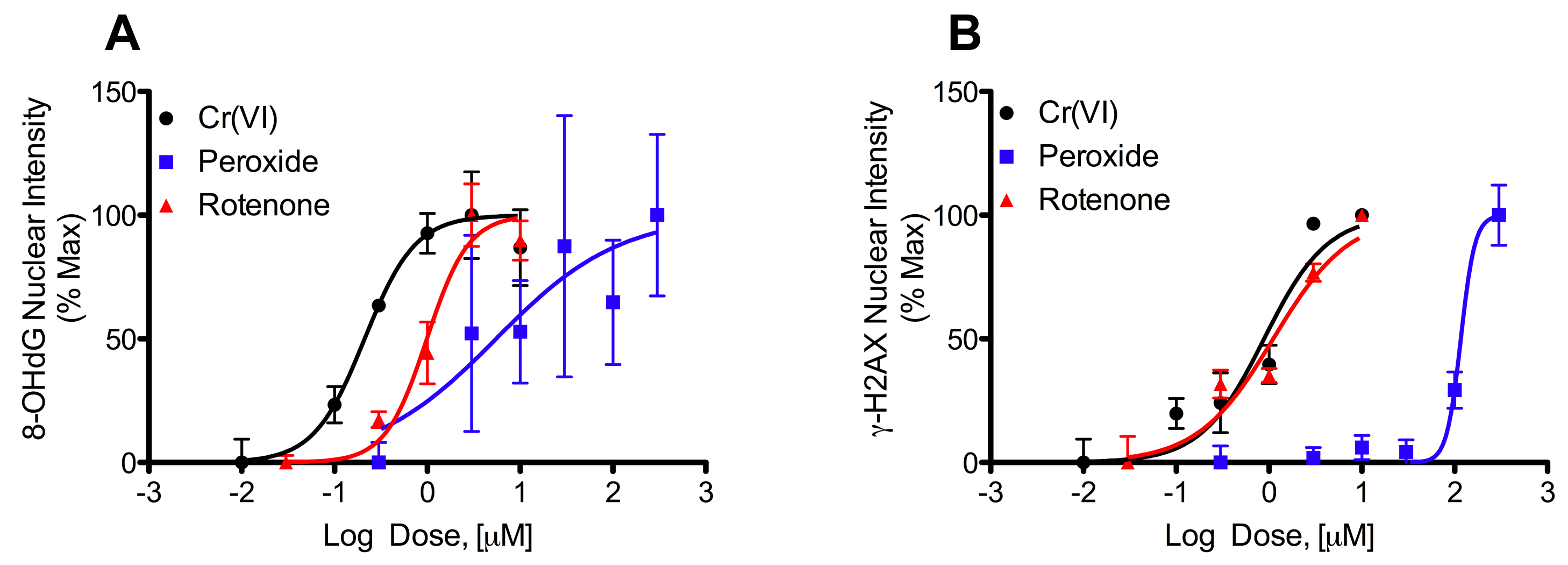

Supplement: Figure S1 — Modeling of nuclear staining intensity in proliferating Caco-2 cells at 24 hr. Concentrations shown include up to first two toxic concentrations. (TIF) [file pone.0042720.s001.tif]
